# Thrombotic adverse events associated with TNF-alpha blockers: a real-world pharmacovigilance analysis of the FAERS database

**DOI:** 10.3389/fphar.2025.1512806

**Published:** 2025-04-25

**Authors:** Lijuan Song, Nan Yang, Xinglong Xing, Yicong Zhao, Jingkai Di

**Affiliations:** ^1^ Department of General Surgery, The Second Affiliated Hospital of Shanxi Medical University, Taiyuan, Shanxi, China; ^2^ Department of Orthopedics, The Third Hospital of Shanxi Medical University, Shanxi Bethune Hospital, Shanxi Academy of Medical Sciences Tongji Shanxi Hospital, Taiyuan, Shanxi, China; ^3^ School of Clinical Medicine, The Second Affiliated Hospital of Shanxi Medical University, Taiyuan, Shanxi, China; ^4^ Department of Orthopedics, The Second Affiliated Hospital of Shanxi Medical University, Taiyuan, Shanxi, China

**Keywords:** TNF-α blockers, thrombosis, FAERS database, adverse event, adalimumab, infliximab

## Abstract

**Objective:**

This research is designed to explore the connection between tumor necrosis factor-α (TNF-α) blocker drugs and thrombotic adverse events.

**Methods:**

The study included data from the FDA Adverse Event Reporting System (FAERS) spanning from the first quarter of 2004 to the first quarter of 2024. We employed the disproportional analysis approach to analyze the signals of thrombosis-related adverse events associated with TNF-α blockers. Moreover, subgroup analyses were conducted to investigate the circumstances of different age and gender groups. Additionally, the induction time and Weibull distribution were utilized for the further interpretation of the data.

**Results:**

During the study period, among 1,382,627 patients in the FAERS database who had adverse events linked to TNF-α inhibitors, 9,714 could be attributed to thrombosis-related adverse events. In the remaining patients, different types of infection events accounted for a large proportion of the proportion. (N = 165,765) Thrombosis-related adverse event signals were detected in all five types of TNF-α inhibitor drugs. Among them, in the analysis of adalimumab, the adverse event signal of postpartum thrombosis was the strongest, and the positive signal of axillary vein thrombosis was the weakest. The analysis based on gender subgroups discovered some positive signals of adverse events that were not observed in the overall population. The Weibull distribution analysis indicated that all five drugs exhibited an premature aging type characteristic, and their induction decreased gradually over time.

**Conclusion:**

This study suggests that TNF - α blockers are associated with various adverse events of thrombosis, with different risks in different patient groups and treatment stages. Clinical doctors should assess individual thrombosis risk and closely monitor coagulation related indicators when using TNF - α inhibitors. This study offers valuable insights for optimizing treatment and improving safety.

## 1 Introduction

Tumor necrosis factor-α (TNF-α) is an inflammatory cytokine that selectively acts on various cell types, and it is expressed and released by macrophages, monocytes, and natural killer cells (Horiuchi et al., 2010; Ruiz et al., 2021). At the structural aspect, it is composed of 157 amino acids. Initially, it exists in the form of a transmembrane molecule (tmTNF) and is catalytically transformed into solTNF under the action of TNF-α converting enzyme (TACE). Both forms of TNF-α possess biological activity and interact with two receptors with distinct affinities to carry out their functions (Schlöndorff et al., 2000). The underlying molecular mechanisms related to TNF-α are intricate, and its biological actions are pleiotropic. A study in which TNF-α activity enhancement treatment was implemented found that TNF-α accelerates the death of tumor cells (Valencia et al., 2006). Besides its therapeutic role, a study that triggers excessive production of TNF-α through activation and accumulation approaches has indicated that it can also cause disease progression and give rise to a series of adverse outcomes such as severe inflammatory lesions, etc (Yiu et al., 2012).

The upsurge of exploration in the complex domain of TNF-α pathway transduction has enabled the expansion and application of corresponding blockers. Currently, the clinically approved TNF-α inhibitors are: infliximab, etanercept, adalimumab, certolizumab pegol, and golimumab. All of them can specifically bind to TNF-α, prevent its interaction with the TNF-α receptor, and have manifested remarkable efficacy in multiple autoimmune diseases (Corrado et al., 2017). A multicentric study demonstrated that adalimumab achieved therapeutically meaningful efficacy in the treatment of noninfectious uveitis (Jaffe et al., 2016); a systematic review research discovered that the clinical outcomes of patients administered infliximab were improved (Chaiyanarm et al., 2024).

As the application of TNF-α blocker drugs becomes widespread, the number of adverse event reports concerning them shows an increasing trend. The five most frequently reported types of adverse events related to TNF-α blockers are as follows: infections and infestations (23.0%), musculoskeletal and connective tissue disorders (28.6%), gastrointestinal disorders (15.3%), skin and subcutaneous tissue disorders (13.5%), and nervous system disorders (11.0%) (Li et al., 2023). Furthermore, recent research has revealed that a relatively long treatment period is commonly needed to obtain satisfactory therapeutic efficacy when employing TNF-α blocker drugs. In a study encompassing 956 cases of Crohn’s disease, the median treatment time of infliximab was as long as 25 months (Hiroz et al., 2014). In a retrospective study focusing on 104 patients with ulcerative proctitis, nearly two-thirds of the patients needed more than 2 years of anti-tumor necrosis factor therapy (Pineton de Chambrun et al., 2020). During this process, the related issues of blood system diseases, especially thrombosis, corresponding to the long-term medication regimens have gradually emerged. Regarding this perspective, multiple relevant cases have been reported. For instance, recurrent acute coronary syndrome was triggered in a patient after infusion of infliximab (Rebolledo Del Toro et al., 2023), and pulmonary embolism occurred in a patient after infusion of infliximab (Bala et al., 2020). This has aroused our concern about the relationship between TNF-α blockers and potential thrombotic risks.

Although there are currently cohort and case studies on thrombosis related to TNF-α blockers, the data have not been updated in detail and the number of reports is relatively limited. A controlled study based on 25 patients within the age range of 40–82 years indicated that the risk of thrombosis would decrease when patients were exposed to TNF-α blocker drugs.And it is consistent with the analysis results of a study involving more than 40 rheumatoid arthritis(RA) patients (Manfredi et al., 2016). However, a study based on 11,881 patients treated with TNF blockers presented a different outcome, namely, that the risk of thrombosis did not increase when patients were exposed to the factor of TNF-α blockers (Davies et al., 2011). Additionally, the results of a case analysis study indicated that TNF-α blockers might increase the risk of thrombosis (Grange et al., 2005), but there is insufficient evidence to prove a direct causal relationship (Livia et al., 2024). The emergence of different results might be attributed to the variations in study design and the population. Therefore, the thrombosis issue related to TNF-α blockers remains an ongoing topic of contention. It must be emphasized that once thrombosis forms, it severely threatens the quality of life of patients. The one-year mortality rate of patients with deep vein thrombosis in Italy is astonishingly as high as 15.4% (Monreal et al., 2019). To sum up, in order to further elucidate the connection between this serious adverse event and TNF-α blockers, a retrospective study based on the FAERS database was conducted in this paper, and then the related thrombosis reports of the five FDA-approved TNF-α blocker drugs, namely, infliximab, etanercept, adalimumab, certolizumab pegol and golimumab, were analyzed.

It is notable that the data from systematic reviews are mainly utilized to support the published articles, and they do not always offer a sufficient dataset for drug research. The FDA Adverse Event Reporting System (FAERS), encompassing tens of millions of adverse events(AE) reports voluntarily submitted by healthcare professionals, consumers, and manufacturers among others, is a public, professional, and free database in the United States. It aims to support the FDA’s safety monitoring of post-marketed drugs and biological products and has achieved breakthroughs in multiple fields of drug research. For instance, the newly marketed drug Esketamine has been discovered to have potential AE and risks in clinical applications, which are mainly manifested in aspects such as long-term efficacy, addiction risk, and suicide risk (Jiang et al., 2023). Additionally, it offers a distinctive reference value for the early identification of the risks of tendinitis and tendon rupture induced by fluoroquinolone drugs that have been utilized in clinical practice for numerous years (Shu et al., 2022). Based on the mining of the FAERS database, it is possible to capture the dynamics of real-world research more accurately. This study aims to assess the risk and severity of thrombosis induced by TNF-α blocker drugs through real-world data and to provide drug vigilance and suggestions for clinical drug safety.

## 2 Materials and methods

### 2.1 Data sources

In this study, data from seven subset files submitted in the open-source FAERS database from the first quarter of 2004 to the first quarter of 2024 were extracted and included: patient demographic and administrative information (DEMO), drug information (DRUG), adverse event coding (REAC), patient outcomes (OUTC), reporting source (RPSR), start and end dates of treatment for the reported drugs (THER), and indications for drug administration (INDI). It encompasses all relevant adverse event and medication error information collected by the FDA. This database adheres to the International Safety Reporting Guideline issued by the International Conference on Harmonisation(ICH E2B) and is updated on a quarterly basis. It elaborately recording the AE parameters related to PRIMARYID, CASEID and CASEVERSION corresponding to the time nodes.

### 2.2 Procedures

**FIGURE 1:**
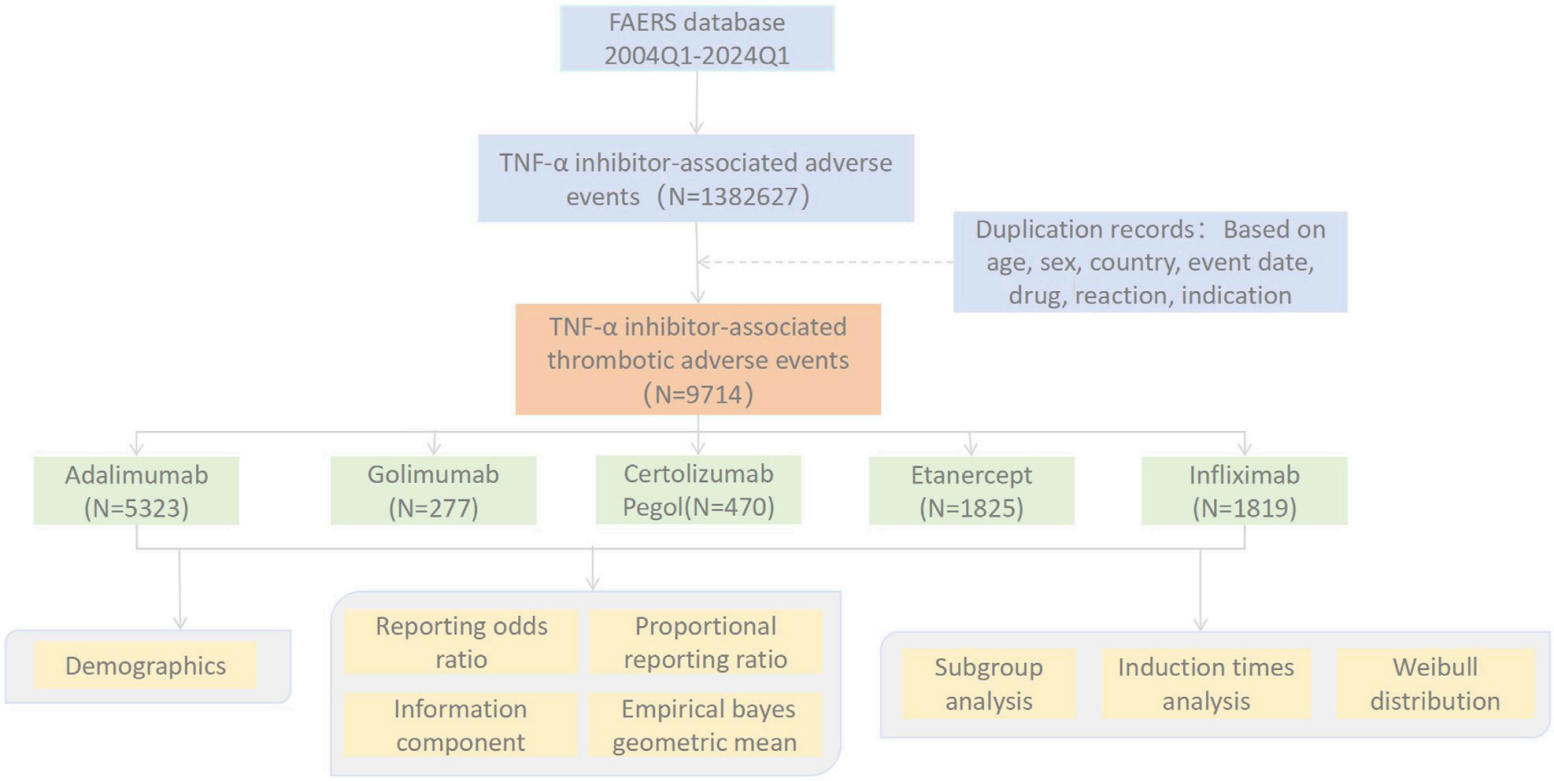
Flow chart showing the analysis process of the study.

To better standardize the adverse event records in the FAERS database, we employed version 26.1 of the Medical Dictionary for Regulatory Activities (MedDRA) to categorize and analyze the retrieved adverse events. Among them, preferred terms (PT) in MedDRA can be designated as higher-level terms (HLT), higher-level group terms (HLGT), and system organ classes (SOC). To enhance the accuracy of result hits, we will primarily focus on the reports where the role_cod of the drug in the file is “PS (primary suspected)”, and the data of different dimensions will be matched according to PRIMARYID. The generic and trade names of the five TNF-α blocker drugs were screened through MeSH subject terms to ensure the recall rate of TNF-α blocker drug names. Furthermore, this paper collated the percentages of severe consequences related to TNF-α blocker drugs and the corresponding cases, as well as the onset time of PT for the identified signals. Finally, in accordance with the recommended guidelines of the FDA, we eliminated reports with recording errors, lacking specific data, and duplicates to reduce bias. The multi-step process of data extraction, processing, and analysis is illustrated in Figure 1.

### 2.3 Data mining

The disproportionality analysis method is employed for the detection of pharmacovigilance signals. The adverse event signals are defined and identified by means of four methods: the reporting odds ratio (ROR), the proportional reporting ratio (PRR), the information component (IC), and the empirical Bayes geometric mean(EBGM), and the specific calculation formula is shown in Supplementary Table S1. In this study, reports that meet the positive signal value in any of the four methods are regarded as meaningful adverse event reports. When a targeted drug is more likely to induce adverse events than all other drugs, it obtains a higher adverse event signal value. Among them, the reporting odds ratio is regarded as a more robust method as it is considered to be capable of reducing bias in estimating the relative risk compared to other methods such as the proportional reporting ratio (Rothman et al., 2004).

After the implementation of the above steps, we conducted analyses on the baseline data and subgroup data, and employed the Kolmogorov-Smirnov method to conduct normality tests for the induction times corresponding to the five drugs. When the data type obeys a normal distribution, parametric tests such as the analysis of variance are adopted. However, when it is a skewed distribution, non-parametric tests such as the rank sum test are employed for the analysis of the data. Furthermore, the Weibull distribution analysis was employed to account for the changing trend of the incidence rate of adverse events over time. All data processing and statistical analyses were performed using R 4.4.1 and Rstudio.

## 3 Results

### 3.1 Descriptive analysis

During the time span from the first quarter of 2004 to the first quarter of 2024, the FAERS database contained patient information on a total of 1,382,627 adverse events related to TNF-α blockers (Table 1). Among the reported patients, the type of TNF-α blocker with the highest frequency of reporting was adalimumab (n = 594,416, 43.0%), and the least was golimumab (n = 43,845, 3.2%). Among the patients related to certolizumab pegol, the proportion of female patient events reached 74.3% (n = 53,737), being the highest among the five drugs. In contrast, within the range of patients taking infliximab, the proportion of female patients was relatively low, at only 44.7% (n = 75,769). In addition, when focusing on the age ratio, patients aged 18–65 accounted for 56.4%.

**TABLE 1:** The clinical distribution characteristics of adverse events reported for five TNF-α inhibitors.

Characteristic	Adalimumab (N, %)	Golimumab (N, %)	Certolizumab pegol (N, %)	Etanercept (N, %)	Infliximab (N, %)	All tumor necrosis factor-α inhibitor (N, %)
Number of patients experiencing adverse reactions	594,416	43,845	72,344	502,466	169,556	1,382,627
Gender						
Female	381,842 (64.2)	28,518 (65.0)	53,737 (74.3)	346,138 (68.9)	75,769 (44.7)	886,004 (64.1)
Male	188,102 (31.6)	11,855 (27.0)	16,032 (22.2)	123,053 (24.5)	52,758 (31.1)	391,800 (28.3)
Missing	24,472 (4.1)	3,472 (7.9)	2,575 (3.6)	33,275 (6.6)	41,029 (24.2)	104,823 (7.6)
Age (year)						
<18	10,016 (1.7)	265 (0.6)	774 (1.1)	10,378 (2.1)	12,265 (7.2)	33,698 (2.4)
18–85	307,723 (51.8)	26,508 (60.5)	36,570 (50.5)	333,204 (66.3)	75,844 (44.8)	779,849 (56.4)
>85	2015 (0.3)	283 (0.6)	227 (0.3)	2,509 (0.5)	395 (0.2)	5,429 (0.4)
Missing	274,662 (46.2)	16,789 (38.3)	34,773 (48.1)	156,375 (31.1)	81,052 (47.8)	563,651 (40.8)
OCCP_COD						
Consumer	433,292 (72.9%)	19,145 (43.7%)	31,039 (42.9%)	190,460 (37.9%)	39,786 (23.5%)	713,722 (51.6%)
Health professional	20,610 (3.5%)	5,902 (13.5%)	12,625 (17.5%)	10,051 (2.0%)	37,302 (22.0%)	86,490 (6.3%)
Lawyer	129 (0.0%)	6 (0.0%)	19 (0.0%)	56 (0.0%)	95 (0.1%)	305 (0.0%)
Physician	67,040 (11.3%)	8,007 (18.3%)	11,608 (16.0%)	221,185 (44.0%)	50,827 (30.0%)	358,667 (25.9%)
Other health-professional	24,368 (4.1%)	5,932 (13.5%)	9,920 (13.7%)	56,024 (11.1%)	35,272 (20.8%)	131,516 (9.5%)
Pharmacist	11,885 (2.0%)	4,206 (9.6%)	5,970 (8.3%)	13,448 (2.7%)	5,079 (3.0%)	40,588 (2.9%)
Registered nurse	80 (0.0%)	4 (0.0%)	64 (0.1%)	58 (0.0%)	26 (0.0%)	232 (0.0%)
Missing	37,012 (6.2%)	643 (1.5%)	1,099 (1.5%)	11,184 (2.2%)	1,169 (0.7%)	51,107 (3.7%)

*N*, number of adverse event reported.

### 3.2 Disproportionality analysis

**FIGURE 2:**
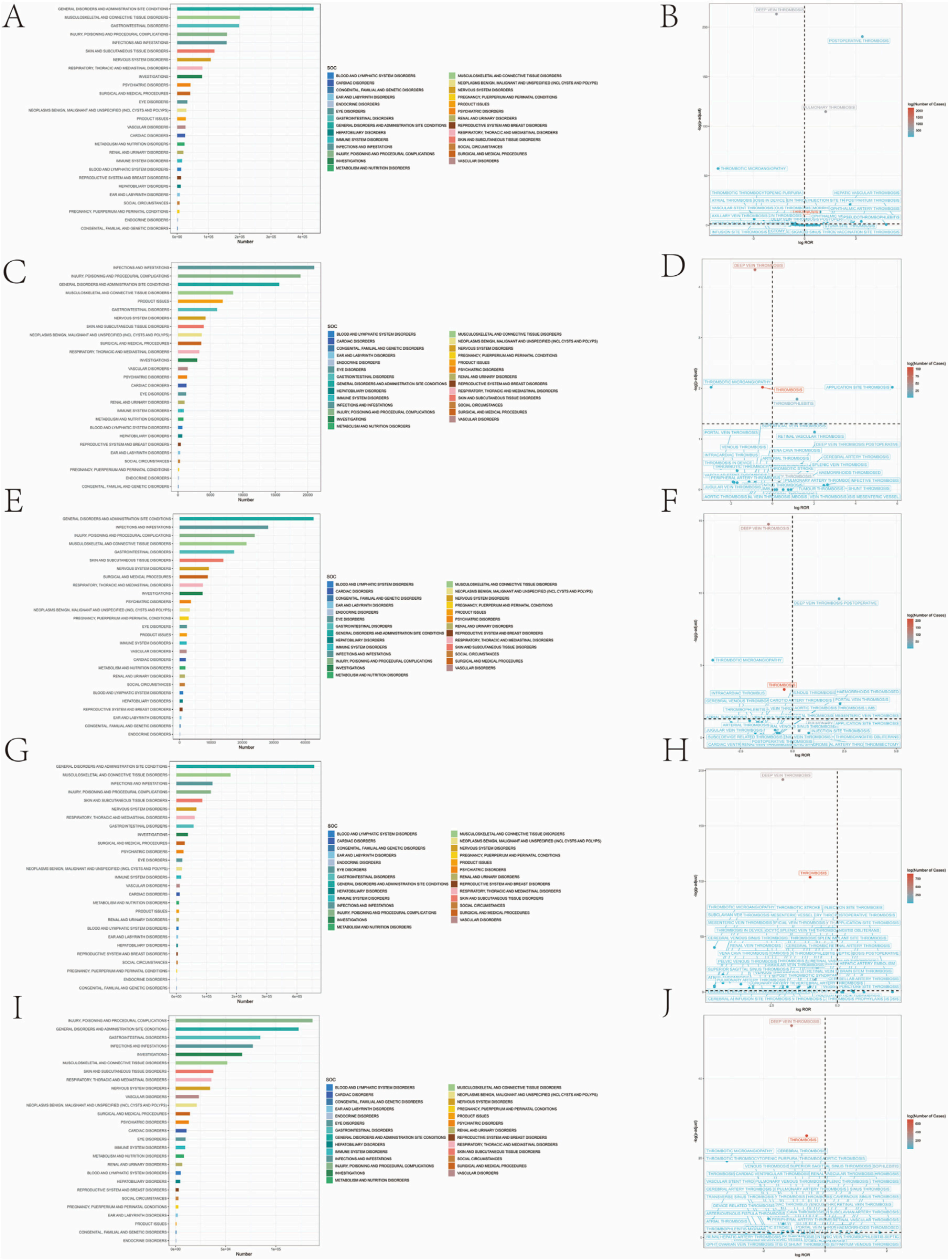
Bar plot and volcanic map. **(A,C,E,G,I)** Bar plot depicting the distribution of thrombosis-related adverse events for Adalimumab, Golimumab, Certolizumab Pegol, Etanercept and Infliximab at the SOC level, respectively. **(B,D,F,H,J)** Volcanic map of difference risk signal for Adalimumab, Golimumab, Certolizumab Pegol, Etanercept, respectively. ROR reporting odds ratios; P.adj, the *p*-value is adjusted with false discovery rate method. SOC, System Organ Class.

Our statistical analysis indicated that a total of 27 organ systems were influenced by the adverse events related to the five TNF-α blockers (Supplementary Table S2; Figure 2). At the SOC level classification, we were informed that among the patients reporting these adverse events, the category of General Disorders and Administration Site Conditions had the highest proportion (1,074,187, 77.7%), while Congenital, Familial and Genetic Disorders ranked at the bottom in terms of magnitude (4,369, 0.3%). Among them, 15 SOCs were related to thrombosis.

**FIGURE 3:**
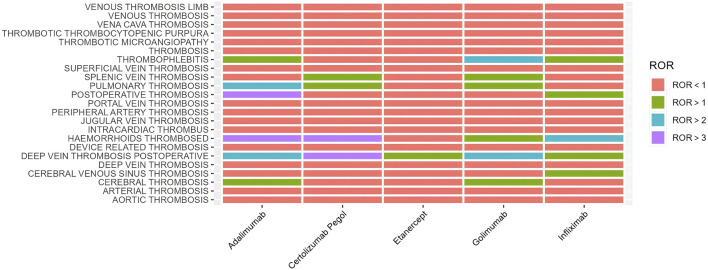
Heatmap of thrombosis-related adverse events that all five TNF-α blockers have reported based on Reporting Odds Ratio at the PT level. PT, Preferred Term.

When concentrating on the five drug-related thrombotic adverse events, our research discovered that in terms of the frequency of occurrence, the drug type most frequently reported for thrombosis formation was adalimumab (n = 5,323), accounting for 54.8% of the total number of thrombosis-related events among the five drug types. The one with the least reporting frequency was golimumab (n = 277), accounting for only 2.9%. And deep vein thrombosis was the most frequently reported type of adverse thrombotic event for golimumab, infliximab, certolizumab pegol and etanercept. Furthermore, in terms of signal intensity, the disproportionality analysis method was employed in this study for data mining and analysis of the results. Among them, in the data analysis of adalimumab, the drug with the largest number of thrombosis events, the adverse event signal of postpartum thrombosis was the strongest (ROR: 36.45, 95% CI: 8.16–162.86), and the positive signal of axillary vein thrombosis was the weakest (ROR: 0.08, 95% CI: 0.01–0.60). Our study used a visual representation called “volcano map” to explore adverse effects in the PT range (Figure 2). Interestingly, although no type of thrombosis was found to have an adverse event signal value meeting the positive criterion with all five drugs in the data of this study, there were significant positive signals of adverse events for postoperative deep vein thrombosis events with the other four drugs except infliximab (Table 2; Figure 3).

**TABLE 2:** Positive signals associated with thrombosis at the PT level of five TNF - α blockers.

PT	N	ROR(95%CI)	PRR(χ2)	EBGM(EBGM05)	IC(IC025)
Adalimumab					
Pulmonary thrombosis	741	2.35(2.18–2.54)	2.35(530.25)	2.24(2.11)	1.17 (-0.5)
Cerebral thrombosis	163	1.88 (1.61–2.21)	1.88 (63.09)	1.83 (1.6)	0.87 (-0.8)
Postoperative thrombosis	144	10.39 (8.57–12.58)	10.39 (885.18)	7.8 (6.64)	2.96 (1.29)
Deep vein thrombosis postoperative	67	2.14 (1.67–2.74)	2.14 (37.6)	2.05 (1.67)	1.04 (-0.63)
Hepatic vascular thrombosis	28	6.33 (4.19–9.54)	6.33 (101.95)	5.32 (3.78)	2.41 (0.73)
Haemorrhoids thrombosed	26	3 (2–4.5)	3 (31.22)	2.8 (2)	1.49 (-0.19)
Injection site thrombosis	25	2.71 (1.8–4.09)	2.71 (24.58)	2.56 (1.81)	1.35 (-0.32)
Ophthalmic vein thrombosis	15	2.18 (1.29–3.69)	2.18 (8.88)	2.09 (1.35)	1.07 (-0.61)
Renal vascular thrombosis	12	2.52 (1.4–4.56)	2.52 (10.1)	2.39 (1.46)	1.26 (-0.42)
Splenic thrombosis	10	2.17 (1.14–4.13)	2.17 (5.84)	2.08 (1.22)	1.06 (-0.62)
Ophthalmic vascular thrombosis	6	6.56 (2.69–15.99)	6.56 (22.81)	5.48 (2.6)	2.46 (0.71)
Vascular pseudoaneurysm thrombosis	5	7.19 (2.69–19.27)	7.19 (21.11)	5.9 (2.59)	2.56 (0.8)
Postpartum thrombosis	4	36.45 (8.16–162.86)	36.45 (59.1)	16.19 (4.63)	4.02 (2.06)
Ophthalmic artery thrombosis	4	9.94 (3.17–31.22)	9.94 (23.59)	7.56 (2.9)	2.92 (1.1)
Thrombosis with thrombocytopenia syndrome	3	3.42 (1.03–11.35)	3.42 (4.56)	3.15 (1.15)	1.65 (-0.1)
Vaccination site thrombosis	1	13.67 (1.24–150.74)	13.67 (7.83)	9.45 (1.27)	3.24 (1.04)
Pseudothrombophlebitis	1	27.34 (1.71–437.06)	27.34 (12.69)	14.17 (1.39)	3.82 (1.47)
Golimumab					
Thrombophlebitis	15	2.27 (1.37–3.78)	2.27 (10.65)	2.27 (1.48)	1.18 (-0.49)
Deep vein thrombosis postoperative	5	2.65 (1.1–6.38)	2.65 (5.11)	2.64 (1.27)	1.4 (-0.27)
Retinal vascular thrombosis	4	4.07 (1.52–10.88)	4.07 (9.18)	4.04 (1.77)	2.02 (0.34)
Cerebral artery thrombosis	3	3.21 (1.03–10)	3.21 (4.54)	3.2 (1.24)	1.68 (0.01)
Application site thrombosis	1	54.13 (6.86–427.25)	54.13 (46.93)	48.81 (8.66)	5.61 (3.73)
Shunt thrombosis	1	5.54 (0.77–39.74)	5.54 (3.67)	5.48 (1.05)	2.46 (0.76)
Infective thrombosis	1	6.25 (0.87–44.9)	6.25 (4.35)	6.18 (1.19)	2.63 (0.93)
Certolizumab Pegol					
Deep vein thrombosis postoperative	17	4.74 (2.93–7.66)	4.74 (49.25)	4.67 (3.13)	2.22 (0.56)
Haemorrhoids thrombosed	5	4.9 (2.02–11.88)	4.9 (15.23)	4.83 (2.3)	2.27 (0.6)
Application site thrombosis	1	28.1 (3.56–221.83)	28.1 (23.52)	25.39 (4.51)	4.67 (2.79)
Etanercept					
Deep vein thrombosis postoperative	34	1.46 (1.04–2.05)	1.46 (4.71)	1.44 (1.08)	0.53 (-1.14)
Application site thrombosis	1	4.24 (0.54–33.47)	4.24 (2.23)	3.92 (0.7)	1.97 (0.09)
Infliximab					
Thrombophlebitis	67	1.42 (1.12–1.81)	1.42 (8.17)	1.41 (1.15)	0.5 (-1.17)
Retinal vein thrombosis	16	1.7 (1.03–2.79)	1.7 (4.47)	1.68 (1.11)	0.75 (-0.92)
Retinal vascular thrombosis	14	2 (1.18–3.41)	2 (6.81)	1.97 (1.26)	0.98 (-0.69)
Haemorrhoids thrombosed	8	2.1 (1.04–4.25)	2.1 (4.49)	2.07 (1.15)	1.05 (-0.63)
Subclavian artery thrombosis	5	3.42 (1.39–8.4)	3.42 (8.15)	3.3 (1.56)	1.72 (0.03)
infective thrombosis	5	4.53(1.83–11.21)	4.53(12.88)	4.31(2.02)	2.11(0.41)

PT, preferred terms; N, number of adverse event reported; ROR, reporting odds ratio; PRR, proportional reporting ratio; EBGM, empirical Bayes geometric mean; IC, information component; CI, confidence interval; 95% CI, two-sided for ROR; *χ2*, chi-squared; EBGM05 and IC025, lower one-sided for EBGM, and IC, respectively.

Based on the stratification methods of gender, this study detected and compared the thrombosis formation signals of five drugs in different subgroups to investigate the potential associations between drugs and adverse thrombotic events in the population (Supplementary Table S3). Among patients treated with adalimumab accompanied by the largest number of adverse events, the positive signal of the female population was stronger than that of the male population in terms of adverse events such as thrombosis of hemorrhoids (ROR: 3.66, 95% CI: 2.22–6.01) and Ophthalmic Artery Thrombosis (ROR: 11.48, 95% CI: 2.87–45.9). However, the male population showed higher positive signal intensities than the female population for other thrombosis types such as cerebral thrombosis (ROR: 2.39, 95%CI: 1.86–3.08), pulmonary artery thrombosis (ROR: 2.67, 95%CI: 2.36–3.02), postoperative thrombosis (ROR: 11.69, 95%CI: 8.44–16.18), hepatic vascular thrombosis (ROR: 8.48, 95%CI: 4.48–16.05), and injection site thrombosis (ROR: 3.78, 95%CI: 1.89–7.55). Interestingly, after conducting stratified studies for different sex populations, some positive signals of adverse events that were not observed in the overall population were discovered. For instance, in male patients, such a signal was detected for adalimumab in vascular graft thrombosis (ROR: 3.24, 95%CI: 1.29–8.13), and for certolizumab pegol in pulmonary artery thrombosis (ROR: 1.94, 95%CI: 1.17–3.22) and mesenteric vein thrombosis (ROR: 4.65, 95%CI: 1.74–12.45). Similarly, in female patients, a few similar results emerged as well. For example, in the case of female patients and infliximab, a positive signal that was not detected at the overall level was presented in splenic vein thrombosis (ROR: 2.85, 95%CI: 1.27–6.43).

In each age subgroup designed in this study, the strongest positive signals were all found in patients taking adalimumab (Table 3). Among patients in the age range of less than 18 years old, the strongest positive signal existed between adalimumab and thrombosis with thrombocytopenia syndrome (ROR: 151.45, 95%CI: 15.75–1,456.08); among patients aged 18–65 years old, the most valuable positive signal was presented between adalimumab and vascular pseudoaneurysm thrombosis (ROR: 10.68, 95%CI: 3.71–30.75); while among patients aged over 65 years old, the strongest positive signal was found between adalimumab and postoperative thrombosis (ROR: 12.38, 95%CI: 8.08–18.96).

**TABLE 3:** The age subgroup data of thrombosis adverse events related to five TNF−α inhibitors.

PT	ROR (95%Cl)		
	<18 years	18–65 years	>65 years
Adalimumab			
Brachiocephalic vein thrombosis	8.41 (1.01–69.89)	0.76 (0.19–3.1)	2.02 (0.27–15)
Carotid artery thrombosis	3.37 (0.44–25.48)	0.62 (0.33–1.17)	1.57 (0.7–3.54)
Cerebral thrombosis	2.13 (0.78–5.78)	1.41 (1.08–1.83)	3.46 (2.59–4.63)
Cerebral venous sinus thrombosis	1.48 (0.66–3.34)	0.43 (0.27–0.69)	1.91 (0.6–6.05)
Cerebral venous thrombosis	0.87 (0.28–2.71)	0.29 (0.16–0.51)	0.37 (0.05–2.65)
Deep vein thrombosis	0.44 (0.23–0.85)	0.29 (0.26–0.33)	0.47 (0.4–0.56)
Injection site thrombosis	10.1 (1.18–86.42)	2.78 (1.63–4.74)	4.45 (1.77–11.15)
Intracardiac thrombus	0.42 (0.06–2.99)	0.92 (0.69–1.23)	1.37 (0.91–2.08)
Pelvic venous thrombosis	0.93 (0.23–3.75)	0.18 (0.08–0.38)	0.61 (0.15–2.48)
Peripheral artery thrombosis	1.53 (0.21–11.19)	0.65 (0.43–0.99)	0.53 (0.24–1.19)
Portal vein thrombosis	2.63 (1.07–6.46)	0.56 (0.39–0.79)	0.42 (0.17–1.01)
Postoperative thrombosis	10.1 (1.18–86.42)	9.47 (6.94–12.91)	12.38 (8.08–18.96)
Pulmonary thrombosis	1.71 (0.42–7)	2.03 (1.79–2.29)	2.77 (2.37–3.24)
Thrombophlebitis	0.72 (0.1–5.19)	1.06 (0.83–1.36)	1.5 (1.01–2.21)
Thrombosis	1.43 (0.93–2.21)	0.98 (0.92–1.05)	1.07 (0.97–1.18)
Thrombosis in device	0.67 (0.09–4.84)	0.11 (0.05–0.24)	0.18 (0.06–0.57)
Thrombosis mesenteric vessel	8.41 (1.01–69.89)	1.45 (0.59–3.58)	0.99 (0.14–7.17)
Transverse sinus thrombosis	2.23 (0.7–7.08)	0.07 (0.01–0.52)	2.62 (0.35–19.65)
Venous thrombosis	0.41 (0.06–2.91)	0.59 (0.43–0.81)	0.91 (0.56–1.47)
Venous thrombosis limb	1.58 (0.22–11.55)	0.87 (0.61–1.24)	1.64 (1.08–2.49)
Etanercept			
Deep vein thrombosis	0.13 (0.03–0.51)	0.17 (0.14–0.19)	0.31 (0.25–0.38)
Thrombophlebitis	0.95(0.13–6.85)	0.37 (0.24–0.57)	0.47 (0.23–0.94)
Thrombosis	0.09 (0.01–0.62)	0.49(0.44–0.53)	0.77(0.68–0.86)
Infliximab			
Arterial thrombosis	1.42 (0.19–10.37)	0.56 (0.25–1.24)	1.16 (0.37–3.61)
Cerebral thrombosis	1.03 (0.25–4.16)	0.49 (0.23–1.02)	0.78 (0.25–2.43)
Deep vein thrombosis	0.88 (0.55–1.4)	0.45 (0.39–0.52)	0.71 (0.54–0.93)
Pelvic venous thrombosis	0.45 (0.06–3.24)	0.54 (0.26–1.13)	2.43(0.6–9.8)
Portal vein thrombosis	2.05 (0.75–5.57)	0.94 (0.59–1.49)	1.32 (0.5–3.54)
Pulmonary thrombosis	3.49(1.27–9.62)	0.87 (0.65–1.18)	0.81 (0.47–1.4)
Superficial vein thrombosis	1 (0.25–4.07)	0.29 (0.15–0.56)	0.3 (0.04–2.14)
Thrombophlebitis	0.71 (0.1–5.11)	1.4(0.98–2.01)	1.56 (0.74–3.28)
Thrombosis	1.07 (0.65–1.75)	0.73 (0.64–0.82)	0.85 (0.69–1.04)
Venous thrombosis	1.22 (0.39–3.84)	0.74 (0.45–1.21)	1.68 (0.84–3.36)
Venous thrombosis limb	3.21 (0.77–13.4)	0.48 (0.21–1.07)	0.55 (0.14–2.19)

ROR, reporting odds ratio; CI, confidence interval; 95% CI, two-sided for ROR.

### 3.3 Time-to-onset of TNF-α blocker drugs-associated thrombotic adverse events

**FIGURE 4:**
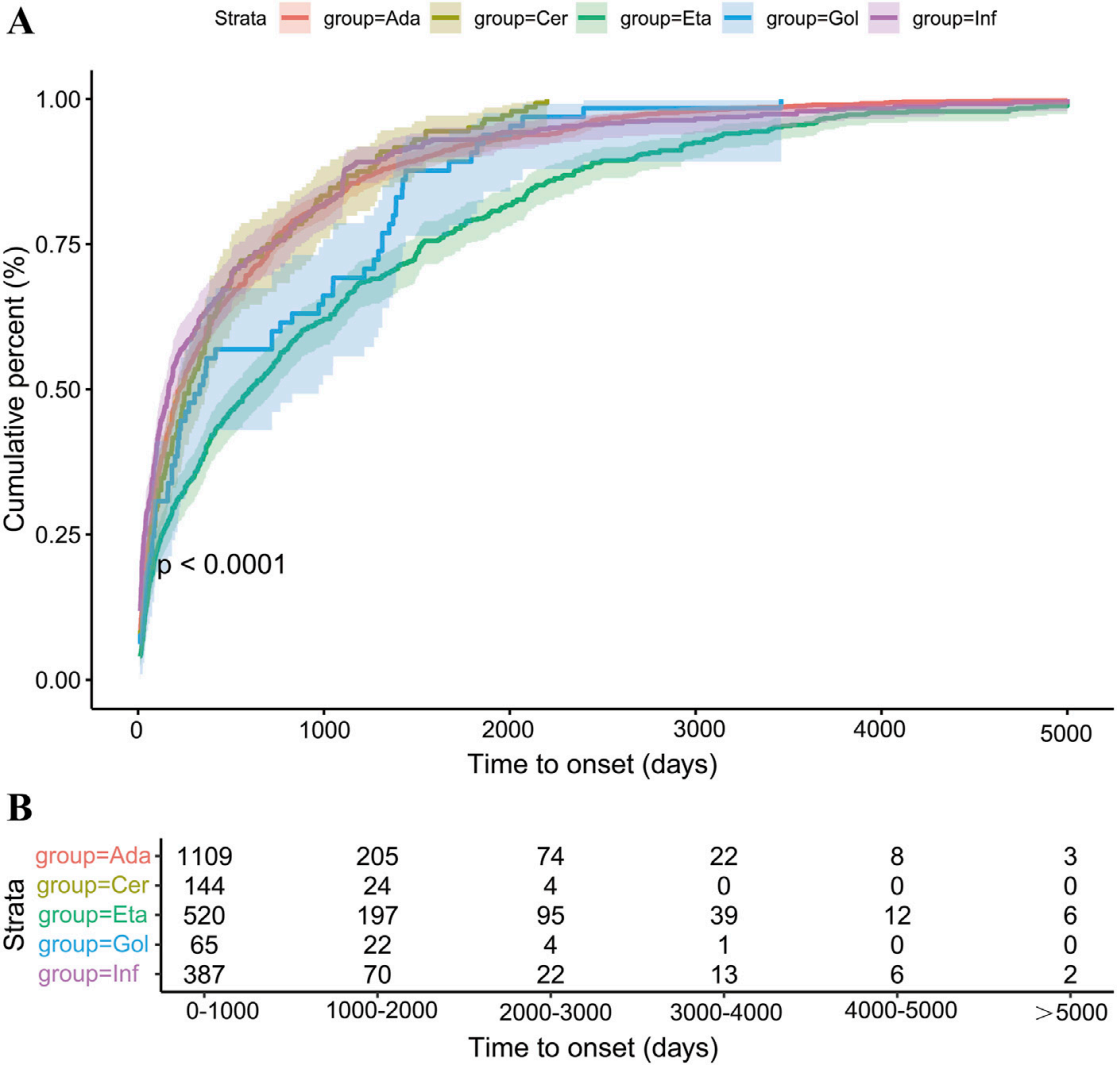
Cumulative distribution function of TNF-α inhibitors by time-to-onset. **(A)** Cumulative incidence curve of TNF-α inhibitors by time-to-onset. The five different color lines indicate the five TNF-α blockers, and the different color areas indicate the 95%CI of the value. **(B)** Distribution numbers of TNF-α inhibitors by time-to-onset.

In our study, a total of approximately 2,225 cases of thrombus-related adverse events reporting the duration of onset were collected, with a median induction time of 278 (Q1 = 69, Q3 = 903) days (Supplementary Table S4). Among them, in the population taking etanercept, the median induction time reached 603.5 (Q1 = 129.75, Q3 = 1,527) days, which was the maximum among the five drugs. In contrast, the median induction time for the population taking adalimumab was only 222 (Q1 = 61, Q3 = 724) days. The results of the K-S method for the corresponding induction times of the five drugs were both *P* < 0.001, which was statistically significant, and the data normality was not satisfied (Figure 4; Supplementary Table S5). Correspondingly, we adopted the method of rank sum test for further analysis. The study demonstrated that there were statistical differences in the overall distribution of the induction times of the five drug groups (H = 100.945, *P* < 0.001). Among them, there were statistical differences between etanercept and three drugs, namely, adalimumab (*P* < 0.001), certolizumab pegol (*P* < 0.001), and infliximab (*P* < 0.001) (Supplementary Table S4). We employed the hazard ratio in the Weibull distribution and the relative increase or decrease in survival time to describe the therapeutic effect. Based on the shape parameter of the survival time, we analyzed the induction time of adverse events in different populations. The results indicated that for these five drugs in the WSP test, the shape parameter β was all less than 1, and the upper limit of its 95% CI was also less than 1 (Table 4).

**TABLE 4:** Weibull parameter test for thrombosis-related adverse events associated with TNF-α inhibitors.

Drugs	Shape parameter (95% CI)	Scale parameter (95% CI)	Type
Adalimumab	0.69 (0.65–0.72)	428.94 (390.07–467.82)	Early failure
Golimumab	0.76 (0.61–0.91)	605.08 (401.13–809.02)	Early failure
Certolizumab Pegol	0.79 (0.69–0.89)	423.69 (331.58–515.80)	Early failure
Etanercept	0.73 (0.68–0.78)	900.26 (788.63–1,011.89)	Early failure
Infliximab	0.60 (0.55–0.65)	354.64 (292.63–416.66)	Early failure

CI, confidence interval; 95% CI, two-sided for shape parameter and scale parameter.

## 4 Discussion

TNF-α blockers are a type of drug employed in the treatment of multiple inflammatory and autoimmune diseases, and are mainly applied in the first-line treatment of rheumatoid arthritis (Hu et al., 2024). Nevertheless, RA patients usually need to use TNF-α blockers for a relatively long period to achieve significant therapeutic outcomes (Seriolo et al., 2006). Under the circumstances of such long-term medication and the patients' own restricted mobility, blood clots are more prone to form within the patients' bodies and they may encounter disastrous and severe setbacks such as pulmonary embolism, myocardial infarction, and cerebral infarction, directly threatening their life and health (Imhof and Koenig, 2001). Hence, it is necessary to investigate the correlation between TNF-α blockers and thrombosis.

This paper investigates five common types of TNF-α blocker drugs in order to monitor more comprehensively the potential risks and efficacies of this class of drugs during clinical use, which offers a meaningful supplement for the choice of medication among different patients. Our results indicate that there is a connection between the administration of TNF-α blockers in the population and the adverse events of thrombosis, which is in line with previous research results. For instance, the treatment with infliximab was discovered to potentially carry the risk of cerebral sinus thrombosis (Tatsuoka et al., 2021). The same situation was also identified in adalimumab (Leblanc et al., 2011). Additionally, we also compared the different tendencies of the five distinct TNF-α blockers regarding thrombosis formation, which is conducive to our better understanding of this type of adverse event.

**FIGURE 5:**
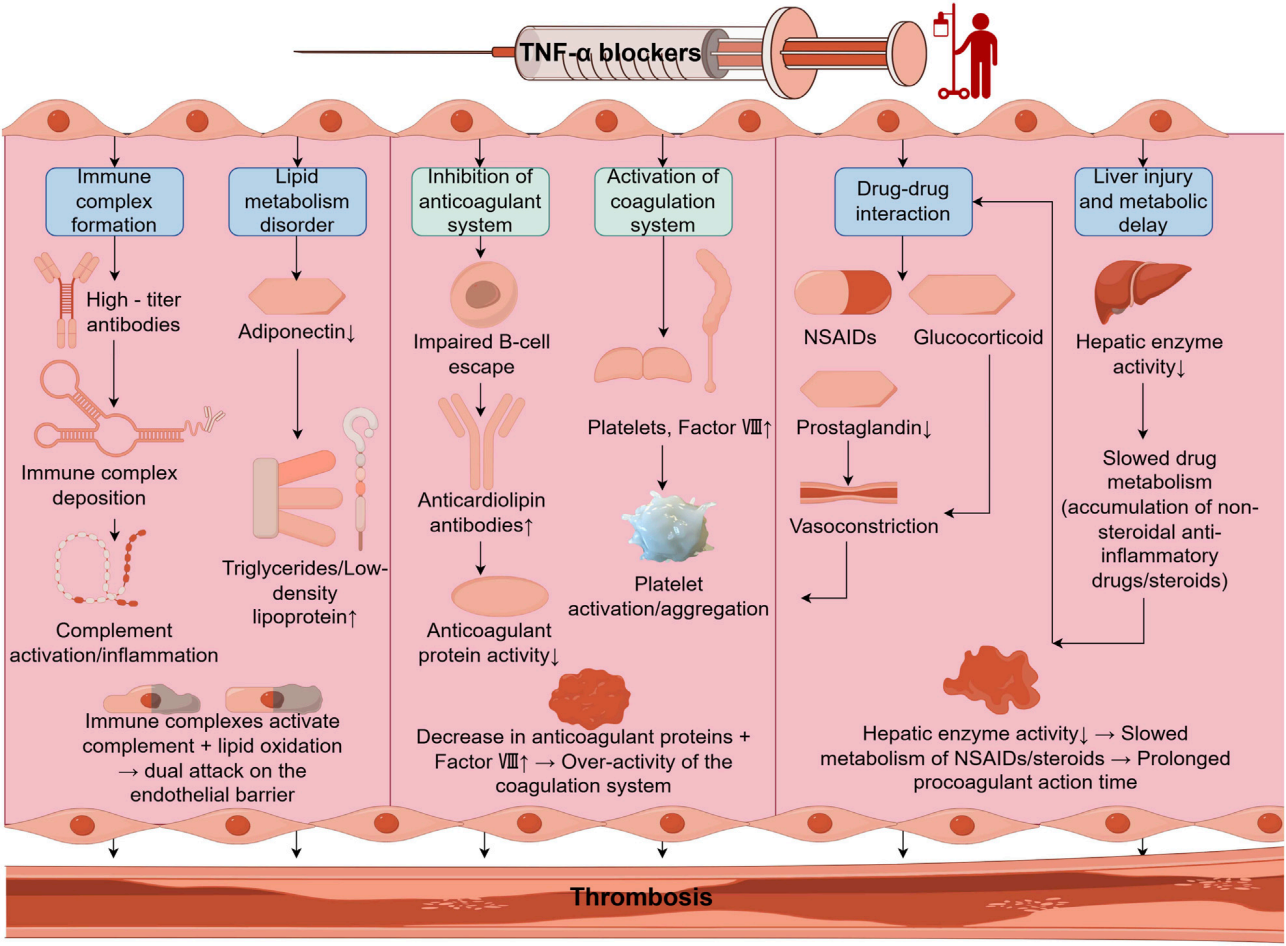
Summary diagram of the mechanism of TNF - α blockers induced thrombosis.

Thrombosis is regarded as a multifactorial disease and there are multiple predisposing factors that synergize with it during its development. Among them, the formation of immune complexes may be an important cause of this adverse event with TNF-α blockers. A study in a randomized, double-blind, placebo-controlled trial showed that in the group of patients using TNF-α inhibitors, 16.7% of the population was detected as positive for the corresponding antibodies, and 93.8% of these patients had a serum antibody concentration titer of more than 1:20, which greatly increased the probability of the immune system recognizing and binding these antibodies, which in turn led to the tendency for a high concentration of immune complexes in the bloodstream, which ultimately resulted in the formation of blood clots (Chung et al., 2003). In addition, adiponectin, as an insulin-sensitizing Hormone, can significantly reduce blood triglyceride and LDL levels, however, its levels were found to be downregulated after TNF-alpha inhibitor treatment, which leads to disruption of lipid metabolism, which exacerbates endothelial dysfunction and ultimately causing thrombosis (Campanati et al., 2015). It is also important to note that TNF-α blockers block the escape of autoimmune B-cells, causing further upregulation of anticardiolipin antibodies, which inhibits the anticoagulant effects of protein C, protein S, and antithrombin III produced downstream of them and promotes hypercoagulability (Yee and Pochapin, 2001; Ferraccioli and Gremese, 2004; Schreiber et al., 2018). Notably, in the group of patients treated with TNF-α blockers, the concentration of platelets and coagulation factor VIII in the blood was increased, which promoted platelet activation and aggregation and exacerbated the imbalance of the coagulation-fibrinolytic system, both of which acted synergistically, ultimately leading to the formation of thrombosis (Di Minno et al., 2014; Bai et al., 2023) (Figure 5).

Additionally, drug interactions constitute another relevant mechanism for thrombosis. Given that the inflammatory environment of patients with rheumatoid arthritis (RA) is often accompanied by the occurrence of multiple symptoms and diseases, multi-drug combination regimens are frequently implemented. For instance, patients with RA are more prone to experiencing pain caused by disordered inflammatory pathways, which results in the concomitant use of pain-relieving drugs such as NSAIDs when they take TNF-α blockers (Smolen et al., 2016). NSAIDs, due to their pharmacological effect of simultaneously inhibiting COX-1 and COX-2 enzymes, reduce the release of prostaglandins, causing an imbalance between prostacyclin and thromboxane A2, and subsequently reinforcing the progression of thrombosis (Zhu et al., 2020). When NSAIDs are combined with TNF-α inhibitors, the possibility of thrombosis is significantly enhanced. Besides, glucocorticoids are another type of drugs that inhibit inflammatory factors. When the organism is chronically exposed to this treatment chronically, the contraction of vascular smooth muscle increases, and vascular resistance rises accordingly. Meanwhile, the risk of thrombosis is also upregulated (Fantidis, 2010). Apart from the above-mentioned superimposed effects, it has been discovered that TNF-α blockers can induce liver injury, resulting in a decline in liver enzyme activity (Penrice et al., 2021; Björnsson et al., 2022). This leads to a slower metabolic rate of the aforementioned drugs in the body, further aggravating the imbalance of the coagulation system in the body and consequently causing thrombosis.

Our research results indicate that although no positive standard adverse event signal value was found between any type of thrombosis and the five drugs in the report, there were such signal values between the adverse event type of postoperative deep vein thrombosis and the other four drugs except infliximab. Compared with other drugs, infliximab was administered more frequently via intravenous injection, while other TNF-α blockers, such as etanercept, adalimumab, and certolizumab pegol, were mainly administered via subcutaneous injection (Jinesh, 2015). The disparity in administration routes can lead to faster onset times, higher absorption rates, and higher clearance rates for intravenous injection, such that the overall drug concentration in the blood is lower than that of subcutaneous injection, resulting in suboptimal exposure (Hemperly and Vande Casteele, 2018). Hence, the positive events of deep vein thrombosis are fewer than those of the others. Furthermore, compared with other TNF-α blockers, infliximab is more capable of enhancing the left ventricular ejection fraction, thereby improving the ejection function and accelerating the blood flow velocity, which reduces the incidence of adverse thrombotic events (Livia et al., 2024). Not only that, its anti-inflammatory effect is also stronger than that of other TNF-α inhibitor drugs in improving cardiovascular function and can prevent tissue damage at an early stage to reduce the stimulation to the coagulation system, which also constitutes an important aspect of its ability to relatively improve the thrombosis situation (Maleki Dizaji et al., 2021).

The results of the gender subgroups indicate that positive signals tend to exhibit a higher intensity in the male subgroup. Furthermore, we also discovered that there were positive signals of adverse events that were not observed in the study of the overall population within the subgroups, and the frequency of such signals in the male subgroup was higher compared to the female subgroup. Differences in physiology, lifestyle, and hormone levels may be the reasons for this outcome. A multicenter multivariate regression analysis study discovered that smoking reduces the therapeutic efficacy of TNF inhibitors (Hyrich et al., 2006). This might cause male patients who smoke more to use TNF-α blockers more frequently in pursuit of higher therapeutic benefits, resulting in a significant occurrence of adverse thrombotic events. Moreover, differences in hormone secretion have contrasting effects on the risk of thrombosis in men and women. It has been discovered that estrogen levels can significantly reduce low-density lipoprotein and cholesterol,and increase high-density lipoprotein levels, thereby reducing the risk of thrombosis (Medina et al., 2003). On the contrary, testosterone leads to an increased possibility of thrombosis through mechanisms such as atherosclerosis models, nitric oxide release-induced vasospasm models (Torrisi et al., 2020). Supplementary, epidemiological studies have shown that males are more prone to certain neurological and psychiatric disorders, such as attention-deficit/hyperactivity disorder (ADHD), compared to females (Erskine et al., 2013). ADHD is a neurodevelopmental disorder with neurobiological complexity. Patients often rely on stimulant drugs for symptom management and frequently suffer from multiple mental health comorbidities. These factors indirectly increase the cardiovascular burden. Ultimately, this leads to a further amplification of the risk of thrombus formation in men compared to women (Luo et al., 2022a; Luo et al., 2022b; Faraone et al., 2024).In the research on different age subgroups, our study demonstrates that the number of thrombosis-related adverse events identified in the adolescent population is lower than that in other age groups and is even missing in groups golimumab and certolizumab pegol. This might be attributed to reporting bias resulting from the less frequent use of TNF-α blocker drugs among minors. Furthermore, for the elderly, they have a higher possibility of thrombosis formation than young adults. Along with the advancement of the aging process of the population, the proportion of highly reactive platelets within the body rises, thereby causing age-related thrombocytosis (Poscablo et al., 2024). Simultaneously, the stability of endothelial cells in the aging state of the body is more prone to being disrupted, resulting in an imbalance in the expression of fibrinolytic factors, which also leads to the occurrence of adverse outcomes (Tofler et al., 2005).

The WSP test indicates that all five types of TNF-α blockers possess premature aging characteristics, and the incidence of their corresponding adverse events gradually decreases over time. This implies that in the actual clinical process, although the use of TNF-α blockers carries the risk of thrombosis formation, for patients who have to use these drugs for a long time, the safety of their long-term use can be guaranteed to a certain extent on the premise of early prevention.

In addition, to distinguish the potential confounding effects of TNF - α inhibitors and the disease itself on thrombosis, we systematically retrieved clinical study data from healthy subjects. Although there is currently no research on whether TNF - α inhibitors cause thrombosis in healthy individuals, some studies suggest that they may increase some risk factors for thrombosis. For example, in some healthy populations with specific metabolic characteristics, clinical trials have shown that TNF - α inhibitors have not improved their basal endothelial function, which may lead to an increased risk of thrombosis. However, further prospective cohort studies are needed to validate these findings.

Our research findings indicate that TNF - α inhibitors have a potential risk of promoting thrombosis, which is of great clinical significance for clinical medication strategies. Doctors should conduct personalized thrombotic risk assessment before prescribing. For patients with high-risk factors such as advanced age, obesity, and hereditary thrombophilia, priority should be given to choosing TNF - α inhibitors with a better thrombotic risk profile (such as infliximab rather than traditional adalimumab). On the other hand, research has shown that existing evidence suggests that tocilizumab (an IL-6 receptor antagonist) exhibits significant therapeutic potential in TNF - α inhibitor resistant patients (Bykerk et al., 2012). And tocilizumab can reduce the level of thrombopoietin, decrease platelet activation, and further reduce the risk of thrombosis (Lupia et al., 2022). This suggests that under strict assessment of bleeding risk, appropriate combination therapy with tocilizumab or antithrombotic drugs may be beneficial in improving patient prognosis and preventing the occurrence of thrombosis. In addition, the results of Weber distribution suggest that although the long-term safety of using TNF - α inhibitors is guaranteed, monitoring of thrombotic events should be strengthened during the high-risk stage of early medication, and a dynamic monitoring system based on thrombotic molecular markers (D-dimer, thrombin antithrombin complex, etc.) should be established.

Our research possesses several advantages. Firstly, disproportionality analysis was employed to monitor new and rare drug safety signals. These findings might allow healthcare professionals and patients to have a more comprehensive understanding of the potential risks of TNF-α blocker drugs, be more cautious when using them, and be able to take preventive measures in advance to control the occurrence of such events. Moreover, through in-depth exploration of the mechanisms and influencing factors of these adverse events, this study will also offer novel ideas and orientations for certain scientific research and drug development. And it can further uncover the mechanisms of drug action and safety concerns, and provide a scientific foundation for the development of safer and more efficacious drugs.

Inevitably, our research also has limitations. First and foremost, the FAERS database is a self-reporting one, which has several inherent selection biases. Moreover, the information in this database depends on the reporters' proficiency, autonomy, and the completeness of their reports. Secondly, there is a lack of data on whether TNF- α inhibitors can cause thrombotic events in healthy populations, which still needs further prospective studies to prove it. Furthermore, although we have exerted every effort to conduct quality control on the data, namely, restricting it to TNF-α blocker drugs as the main suspected drugs, the interference of confounding factors cannot be completely excluded, resulting in a certain extent of bias in the results. Ultimately, this study did not quantify the risks nor infer definite causal relationships but merely provided an estimation of signal strength. Hence, higher-quality and larger-scale prospective studies are still requisite to confirm the causal associations among them, thereby enhancing the credibility of the present conclusions.

## 5 Conclusion

Through the analysis of the information in the FAERS database by employing different strategies, we discovered that there exists a correlation between TNF-α blocker drugs and thrombosis-related adverse events. This research contributes to enhancing the understanding of the safety of TNF-α blockers among medical professionals and further offers valuable insights for the prevention of thrombosis-related adverse events and clinical practice.

## Data Availability

The original contributions presented in the study are included in the article/Supplementary Material, further inquiries can be directed to the corresponding author.
