# Supplementary material for: Thrombotic adverse events associated with TNF-alpha blockers: a real-world pharmacovigilance analysis of the FAERS database
Source: Front Pharmacol. 2025 Apr 25;16:1512806. doi: 10.3389/fphar.2025.1512806 (PMC12061870; doi:10.3389/fphar.2025.1512806)
Supplement: Supplementary file 1 [file Table1.docx]

**Table S1** The formula to calculate ROR(95%CI)，PRR(χ²)，EBGM(EBGM05) and IC(IC025).

| **Name of Algorithm** | **Formula** | **Positive Signal Criteria** |
| --- | --- | --- |
| **ROR** | ROR=ad/bc | Lower Limit of 95%CI>1, N≥3 |
|  | 95%CI=e ^ln(ROR)±1.96(1/a+1/b+1/c+1/d)^0.5^ |  |
| **PRR** | PRR=(a(c+d))/(c(a+b)) | PRR≥2, X²≥4, N≥3 |
|  | *χ2*=[(ad-bc)^2](a+b+c+d)/[(a+b)(c+d)(a+c)(b+d)] |  |
| **BCPNN** | IC=log_2_a(a+b+c+d)(a+c)(a+b) | IC_025_>0 |
|  | IC_025_=e ^ln(IC)-1.96(1/a+1/b+1/c+1/d)^0.5^ |  |
| **MGPS** | EBGM=a(a+b+c+d)/((a+c)/(a+b)) | EBGM05>2, N>0 |
|  | EBGM05=e ^ln(EBGM)-1.64(1/a+1/b+1/c+1/d)^0.5^ |  |

*ROR*, reporting odds ratio; *PRR*, proportional reporting ratio; *EBGM*, empirical Bayes geometric mean; *IC*, information component; *CI*, confidence interval; *95% CI*, two-sided for ROR; *χ2*, chi-squared; *EBGM05 and IC025*, lower one-sided for EBGM and IC, respectively.

**Table S2** Comparison of ROR values for five TNF-α inhibitors related to thrombotic adverse events at the SOC level.

| **SOC** | **Adalimumab** | **Golimumab** | **Certolizumab Pegol** | **Etanercept** | **Infliximab** |
| --- | --- | --- | --- | --- | --- |
| general disorders and administration site conditions | 1.44 (1.44-1.45) | 0.79 (0.77-0.8) | 1.2 (1.19-1.21) | 2.45(2.44-2.46) | 0.87 (0.87-0.88) |
| musculoskeletal and connective tissue disorders | 2.23 (2.22-2.24) | 1.52(1.48-1.55) | 2.04(2.01-2.06) | 2.85(2.83-2.86) | 1.27(1.26-1.28) |
| gastrointestinal disorders | 1.27(1.26-1.27) | 0.62(0.61-0.64) | 0.96(0.95-0.98) | 0.46(0.46-0.46) | 1.29(1.28-1.3) |
| injury, poisoning and procedural complications | 0.8(0.8-0.81) | 1.83(1.8-1.86) | 1.13(1.11-1.14) | 0.8(0.8-0.8) | 1.86(1.85-1.88) |
| infections and infestations | 1.68(1.67-1.69) | 4.27(4.21-4.34) | 2.79(2.75-2.82) | 1.76 (1.75-1.77) | 1.97(1.96-1.99) |
| skin and subcutaneous tissue disorders | 1.2(1.19-1.2) | 0.66(0.64-0.68) | 1.25(1.23-1.27) | 1.19(1.18-1.2) | 0.88(0.88-0.89) |
| nervous system disorders | 0.64(0.63-0.64) | 0.43(0.42-0.45) | 0.5(0.49-0.51) | 0.54(0.54-0.55) | 0.49(0.48-0.49) |
| respiratory, thoracic and mediastinal disorders | 0.9(0.89-0.91) | 0.62(0.6-0.65) | 0.74(0.72-0.75) | 0.94(0.93-0.95) | 0.96(0.95-0.97) |
| investigations | 0.66(0.65-0.66) | 0.42(0.41-0.44) | 0.54(0.53-0.56) | 0.43(0.43-0.44) | 1.4(1.39-1.41) |
| psychiatric disorders | 0.38(0.37-0.38) | 0.21(0.2-0.23) | 0.29(0.28-0.3) | 0.29(0.28-0.29) | 0.28(0.28-0.29) |
| surgical and medical procedures | 1.72(1.7-1.74) | 2.53(2.45-2.62) | 3.35(3.28-3.43) | 1.57(1.55-1.58) | 1.4(1.37-1.42) |

**Table S2** (Continued) Comparison of ROR values for five TNF-α inhibitors related to thrombotic adverse events at the SOC level.

| **SOC** | **Adalimumab** | **Golimumab** | **Certolizumab Pegol** | **Etanercept** | **Infliximab** |
| --- | --- | --- | --- | --- | --- |
| eye disorders | 0.87(0.86-0.88) | 0.58(0.55-0.61) | 0.56(0.53-0.58) | 0.71(0.7-0.72) | 0.64(0.63-0.65) |
| neoplasms benign, malignant and unspecified (incl cysts and polyps) | 0.57(0.57-0.58) | 1.27(1.23-1.31) | 0.57(0.55-0.59) | 0.51(0.5-0.51) | 1.02(1-1.03) |
| product issues | 0.94(0.93-0.95) | 4.21(4.11-4.32) | 0.7(0.67-0.72) | 0.4(0.39-0.41) | 0.08(0.07-0.08) |
| vascular disorders | 0.65(0.64-0.66) | 0.64(0.61-0.68) | 0.49(0.47-0.51) | 0.38(0.37-0.39) | 1.4(1.38-1.41) |
| cardiac disorders | 0.49(0.49-0.5) | 0.45(0.42-0.47) | 0.35(0.34-0.37) | 0.3(0.29-0.3) | 0.52(0.51-0.53) |
| metabolism and nutrition disorders | 0.56(0.55-0.57) | 0.35(0.32-0.37) | 0.4(0.38-0.42) | 0.29(0.28-0.29) | 0.49(0.48-0.5) |
| renal and urinary disorders | 0.53(0.52-0.54) | 0.48(0.45-0.51) | 0.41(0.39-0.43) | 0.31(0.3-0.32) | 0.43(0.42-0.44) |
| immune system disorders | 0.76(0.75-0.77) | 0.75(0.71-0.8) | 0.97(0.93-1.02) | 1.05(1.04-1.07) | 1.12(1.09-1.14) |
| blood and lymphatic system disorders | 0.39(0.39-0.4) | 0.36(0.34-0.39) | 0.32(0.3-0.34) | 0.31(0.31-0.32) | 0.38(0.37-0.39) |
| reproductive system and breast disorders | 0.73(0.71-0.74) | 0.44(0.4-0.49) | 0.5(0.47-0.54) | 0.39(0.38-0.41) | 0.44(0.43-0.46) |

**Table S2** (Continued) Comparison of ROR values for five TNF-α inhibitors related to thrombotic adverse events at the SOC level.

| **SOC** | **Adalimumab** | **Golimumab** | **Certolizumab Pegol** | **Etanercept** | **Infliximab** |
| --- | --- | --- | --- | --- | --- |
| hepatobiliary disorders | 0.66(0.65-0.67) | 0.67(0.62-0.72) | 0.54(0.5-0.57) | 0.39(0.38-0.4) | 0.63(0.62-0.65) |
| ear and labyrinth disorders | 1.05(1.03-1.07) | 0.7(0.63-0.78) | 0.68(0.63-0.73) | 0.99(0.96-1.02) | 0.66(0.63-0.69) |
| social circumstances | 0.88(0.86-0.9) | 0.6(0.54-0.67) | 1.73(1.65-1.81) | 0.76(0.74-0.78) | 0.79(0.76-0.82) |
| pregnancy, puerperium and perinatal conditions | 0.91(0.89-0.93) | 0.37(0.32-0.43) | 3.27(3.15-3.39) | 0.47(0.45-0.49) | 0.81(0.78-0.84) |
| endocrine disorders | 0.59(0.56-0.61) | 0.56(0.48-0.66) | 1.18(0.17-8.43) | 0.38(0.36-0.4) | 0.45(0.42-0.48) |
| congenital, familial and genetic disorders | 0.32(0.31-0.34) | 0.24(0.19-0.29) | 0.47(0.42-0.52) | 0.25(0.23-0.26) | 0.37(0.34-0.39) |

*SOC*, systemic organ classes; *ROR*, reporting odds ratio; *CI*, confidence interval; *95% CI*, two-sided for ROR.

**Table S3** The gender subgroup data of thrombosis adverse events related to five TNF-α inhibitors.

|  | **ROR(95%Cl)** | |
| --- | --- | --- |
|  | **Male** | **Female** |
| **Adalimumab** |  |  |
| aortic thrombosis | 0.67(0.35-1.3) | 0.97(0.6-1.55) |
| arterial thrombosis | 0.57(0.33-0.96) | 0.65(0.42-1.02) |
| atrial thrombosis | 0.17(0.07-0.41) | 0.11(0.03-0.33) |
| basilar artery thrombosis | 0.37(0.05-2.66) | 0.34(0.05-2.43) |
| brachiocephalic vein thrombosis | 2.33(0.72-7.54) | 0.44(0.06-3.19) |
| cardiac ventricular thrombosis | 0.72(0.37-1.39) | 0.46(0.19-1.12) |
| carotid artery thrombosis | 1.74(0.97-3.1) | 0.6(0.32-1.13) |
| catheter site thrombosis | 0.41(0.06-2.98) | 2.32(1.17-4.61) |
| cavernous sinus thrombosis | 0.37(0.05-2.66) | 0.43(0.11-1.75) |
| cerebral artery thrombosis | 0.58(0.18-1.82) | 0.36(0.14-0.97) |
| cerebral thrombosis | 2.39(1.86-3.08) | 1.65(1.34-2.03) |
| cerebral venous sinus thrombosis | 0.69(0.33-1.46) | 0.54(0.37-0.78) |
| cerebral venous thrombosis | 0.48(0.21-1.07) | 0.32(0.19-0.54) |
| coronary artery thrombosis | 0.79(0.54-1.16) | 0.67(0.43-1.06) |
| deep vein thrombosis | 0.4(0.35-0.45) | 0.27(0.25-0.3) |
| deep vein thrombosis postoperative | 1.48(0.36-6.12) | 1.98(1.5-2.61) |
| device related thrombosis | 0.62(0.29-1.31) | 0.4(0.19-0.85) |
| graft thrombosis | 0.8(0.25-2.51) | 0.24(0.03-1.73) |
| haemorrhoids thrombosed | 2.54(1.23-5.22) | 3.66(2.22-6.01) |
| hepatic artery thrombosis | 0.5(0.12-2.01) | 0.29(0.04-2.12) |
| hepatic vascular thrombosis | 8.48(4.48-16.05) | 5.57(3.22-9.61) |
| hepatic vein thrombosis | 1.71(0.69-4.2) | 1.74(0.91-3.31) |
| infective thrombosis | 1.68(0.41-6.97) | 1.43(0.34-5.99) |
| injection site thrombosis | 3.78(1.89-7.55) | 2.42(1.44-4.04) |
| intracardiac thrombus | 0.77(0.54-1.1) | 1.12(0.88-1.42) |
| jugular vein thrombosis | 0.41(0.18-0.91) | 0.33(0.19-0.55) |
| medical device site thrombosis | 1.04(0.14-7.6) | 2.3(0.7-7.52) |
| mesenteric artery thrombosis | 0.19(0.03-1.37) | 0.88(0.36-2.14) |
| mesenteric vein thrombosis | 1(0.56-1.77) | 0.82(0.49-1.37) |
| ophthalmic artery thrombosis | 6.22(0.73-53.22) | 11.48(2.87-45.9) |
| ophthalmic vascular thrombosis | 2.59(0.34-19.92) | 3.53(0.8-15.65) |
| ophthalmic vein thrombosis | 1.94(0.61-6.24) | 2.35(1.3-4.26) |
| pelvic venous thrombosis | 0.64(0.29-1.44) | 0.16(0.08-0.35) |
| peripheral artery thrombosis | 0.86(0.56-1.33) | 0.61(0.4-0.95) |
| portal vein thrombosis | 0.73(0.51-1.05) | 0.73(0.52-1.03) |
| post thrombotic syndrome | 0.57(0.14-2.31) | 0.32(0.12-0.86) |
| postoperative thrombosis | 11.69(8.44-16.18) | 10.74(8.4-13.72) |
| pulmonary artery thrombosis | 0.62(0.28-1.4) | 0.78(0.43-1.43) |
| pulmonary thrombosis | 2.67(2.36-3.02) | 2.08(1.89-2.29) |
| pulmonary venous thrombosis | 0.45(0.06-3.24) | 0.53(0.07-3.88) |
| renal vascular thrombosis | 2.07(0.64-6.67) | 2.87(1.44-5.74) |
| renal vein thrombosis | 0.85(0.35-2.08) | 0.34(0.13-0.91) |
| retinal artery thrombosis | 0.6(0.08-4.32) | 0.71(0.26-1.93) |
| retinal vascular thrombosis | 0.63(0.2-1.96) | 0.74(0.38-1.44) |
| retinal vein thrombosis | 0.98(0.46-2.08) | 0.66(0.35-1.23) |
| splenic thrombosis | 3.81(1.63-8.89) | 1.61(0.58-4.44) |
| splenic vein thrombosis | 0.45(0.14-1.41) | 0.43(0.16-1.16) |
| subclavian artery thrombosis | 1.94(0.47-8.11) | 0.36(0.05-2.63) |
| subclavian vein thrombosis | 0.3(0.1-0.95) | 0.07(0.02-0.28) |
| superficial vein thrombosis | 0.67(0.43-1.05) | 0.29(0.2-0.42) |
| superior sagittal sinus thrombosis | 0.68(0.25-1.84) | 0.05(0.01-0.33) |
| thromboangiitis obliterans | 1.24(0.3-5.11) | 2.04(0.73-5.67) |
| thrombophlebitis | 1.18(0.85-1.62) | 1.11(0.9-1.38) |
| thrombosed varicose vein | 3.11(0.4-24.29) | 0.7(0.1-5.09) |
| thrombosis | 0.94(0.87-1.01) | 0.87(0.82-0.91) |
| thrombosis in device | 0.2(0.11-0.38) | 0.18(0.09-0.35) |
| thrombosis mesenteric vessel | 1.66(0.61-4.53) | 1.73(0.8-3.73) |
| thrombosis prophylaxis | 1.55(0.21-11.58) | 0.96(0.13-7.07) |
| thrombotic cerebral infarction | 0.19(0.03-1.33) | 0.36(0.09-1.47) |
| thrombotic microangiopathy | 0.06(0.03-0.13) | 0.01(0-0.05) |
| thrombotic stroke | 1.69(0.99-2.9) | 0.87(0.5-1.52) |
| thrombotic thrombocytopenic purpura | 0.18(0.09-0.38) | 0.12(0.06-0.24) |
| transverse sinus thrombosis | 1(0.25-4.1) | 0.17(0.06-0.54) |
| tumour thrombosis | 0.29(0.04-2.1) | 0.41(0.06-2.96) |
| vascular stent thrombosis | 0.18(0.08-0.41) | 0.12(0.03-0.47) |
| vena cava thrombosis | 0.59(0.32-1.1) | 0.2(0.09-0.41) |
| venous thrombosis | 0.59(0.39-0.88) | 0.65(0.49-0.86) |
| venous thrombosis limb | 0.98(0.64-1.48) | 0.93(0.68-1.26) |
| visceral venous thrombosis | 2.39(0.31-18.28) | 1.64(0.22-12.47) |
| **Golimumab** |  |  |
| cerebral thrombosis | 2.89(1.08-7.71) | 0.56(0.14-2.22) |
| deep vein thrombosis | 0.79(0.54-1.17) | 0.45(0.33-0.62) |
| intracardiac thrombus | 0.5(0.07-3.57) | 0.26(0.04-1.87) |
| pulmonary thrombosis | 1.35(0.64-2.83) | 1.23(0.77-1.98) |
| retinal vascular thrombosis | 8.84(2.19-35.67) | 2.78(0.69-11.18) |
| superficial vein thrombosis | 0.7(0.1-5) | 0.51(0.16-1.58) |
| thrombophlebitis | 1.86(0.6-5.79) | 2.57(1.46-4.54) |
| thrombosis | 0.57(0.38-0.85) | 0.8(0.64-1) |
| thrombotic microangiopathy | 0.21(0.03-1.48) | 0.12(0.02-0.84) |
| thrombotic thrombocytopenic purpura | 0.54(0.08-3.87) | 0.52(0.13-2.09) |
| venous thrombosis | 1.03(0.26-4.12) | 0.22(0.03-1.55) |
| venous thrombosis limb | 0.88(0.12-6.24) | 0.36(0.05-2.54) |
| **Certolizumab Pegol** |  |  |
| aortic thrombosis | 1.05(0.15-7.45) | 0.41(0.06-2.92) |
| arterial thrombosis | 0.57(0.08-4.04) | 0.25(0.04-1.8) |
| deep vein thrombosis | 0.83(0.61-1.13) | 0.34(0.27-0.44) |
| intracardiac thrombus | 0.34(0.05-2.39) | 0.24(0.06-0.97) |
| mesenteric vein thrombosis | 4.65(1.74-12.45) | 0.84(0.21-3.38) |
| peripheral artery thrombosis | 1.15(0.29-4.59) | 0.68(0.22-2.12) |
| portal vein thrombosis | 1.36(0.51-3.63) | 0.32(0.08-1.29) |
| pulmonary thrombosis | 1.94(1.17-3.22) | 1(0.7-1.44) |
| thrombectomy | 5.1(0.71-36.64) | 0.94(0.13-6.68) |
| thrombosis | 1.02(0.8-1.31) | 0.7(0.6-0.83) |
| thrombotic microangiopathy | 0.14(0.02-0.99) | 0.05(0.01-0.39) |
| vena cava thrombosis | 0.83(0.12-5.9) | 0.22(0.03-1.58) |
| venous thrombosis limb | 1.18(0.29-4.72) | 0.5(0.16-1.54) |
| **Etanercept** |  |  |
| aortic thrombosis | 0.42(0.14-1.31) | 0.2(0.06-0.62) |
| arterial thrombosis | 0.15(0.04-0.61) | 0.21(0.09-0.5) |
| arteriovenous fistula thrombosis | 0.37(0.05-2.61) | 0.26(0.04-1.84) |
| atrial thrombosis | 0.13(0.03-0.52) | 0.05(0.01-0.33) |
| brain stem thrombosis | 3.12(0.42-23.27) | 1.09(0.15-8.05) |
| carotid artery thrombosis | 0.53(0.13-2.12) | 0.15(0.04-0.61) |
| cerebral thrombosis | 0.39(0.17-0.86) | 0.36(0.22-0.57) |
| cerebral venous sinus thrombosis | 0.18(0.03-1.31) | 0.02(0-0.18) |
| cerebral venous thrombosis | 0.3(0.07-1.21) | 0.14(0.06-0.35) |
| coronary artery thrombosis | 0.49(0.26-0.95) | 0.13(0.04-0.42) |
| deep vein thrombosis | 0.39(0.33-0.46) | 0.19(0.17-0.22) |
| deep vein thrombosis postoperative | 1.38(0.19-10) | 1.48(1.04-2.1) |
| hepatic vascular thrombosis | 1.08(0.15-7.78) | 0.36(0.05-2.62) |
| intracardiac thrombus | 0.23(0.09-0.54) | 0.34(0.21-0.54) |
| jugular vein thrombosis | 0.39(0.12-1.2) | 0.24(0.12-0.48) |
| peripheral artery thrombosis | 0.54(0.26-1.13) | 0.33(0.17-0.64) |
| portal vein thrombosis | 0.18(0.07-0.48) | 0.24(0.12-0.46) |
| post thrombotic syndrome | 1.09(0.27-4.4) | 0.31(0.1-0.96) |
| postoperative thrombosis | 0.65(0.16-2.64) | 0.82(0.41-1.66) |
| pulmonary artery thrombosis | 0.19(0.03-1.39) | 0.09(0.01-0.63) |
| pulmonary thrombosis | 0.68(0.49-0.93) | 0.49(0.39-0.6) |
| pulmonary venous thrombosis | 0.86(0.12-6.18) | 0.69(0.09-4.99) |
| renal vascular thrombosis | 1.26(0.17-9.13) | 0.37(0.05-2.65) |
| retinal artery thrombosis | 3.55(1.11-11.39) | 0.45(0.11-1.82) |
| retinal vascular thrombosis | 1.6(0.59-4.32) | 0.31(0.1-0.97) |
| retinal vein thrombosis | 0.52(0.13-2.1) | 0.84(0.45-1.58) |
| splenic vein thrombosis | 0.28(0.04-2.02) | 0.42(0.13-1.3) |
| subclavian artery thrombosis | 1.79(0.25-13.12) | 0.95(0.23-3.89) |
| superficial vein thrombosis | 0.25(0.09-0.67) | 0.19(0.11-0.32) |
| thrombophlebitis | 0.73(0.42-1.26) | 0.4(0.27-0.6) |
| thrombosis | 0.6(0.53-0.67) | 0.47(0.43-0.51) |
| thrombosis in device | 0.04(0.01-0.27) | 0.05(0.01-0.2) |
| thrombotic microangiopathy | 0.07(0.03-0.2) | 0.04(0.01-0.09) |
| thrombotic stroke | 0.44(0.11-1.77) | 0.17(0.04-0.67) |
| thrombotic thrombocytopenic purpura | 0.05(0.01-0.35) | 0.24(0.13-0.41) |
| venous thrombosis | 0.32(0.15-0.68) | 0.18(0.1-0.33) |
| venous thrombosis limb | 0.47(0.21-1.06) | 0.46(0.28-0.74) |
| **Infliximab** |  |  |
| aortic thrombosis | 0.44(0.11-1.77) | 1.59(0.75-3.36) |
| arterial thrombosis | 0.36(0.12-1.11) | 0.98(0.47-2.06) |
| atrial thrombosis | 0.2(0.05-0.81) | 0.16(0.02-1.11) |
| carotid artery thrombosis | 2.1(0.86-5.09) | 0.51(0.13-2.07) |
| cavernous sinus thrombosis | 2.24(0.55-9.12) | 2.85(0.91-8.99) |
| cerebral artery thrombosis | 0.57(0.08-4.08) | 0.39(0.05-2.78) |
| cerebral thrombosis | 0.92(0.48-1.77) | 0.56(0.28-1.13) |
| cerebral venous sinus thrombosis | 3.29(1.8-6.01) | 1.2(0.71-2.02) |
| cerebral venous thrombosis | 1.92(0.95-3.86) | 1.28(0.74-2.21) |
| coronary artery thrombosis | 0.34(0.13-0.92) | 0.6(0.23-1.61) |
| deep vein thrombosis | 0.73(0.63-0.85) | 0.55(0.48-0.64) |
| device related thrombosis | 0.26(0.04-1.85) | 0.25(0.03-1.76) |
| haemorrhoids thrombosed | 1.79(0.44-7.25) | 3.15(1.16-8.51) |
| hepatic vein thrombosis | 1.98(0.49-8.03) | 0.71(0.1-5.07) |
| infusion site thrombosis | 2.42(0.59-9.84) | 2.42(0.77-7.6) |
| intracardiac thrombus | 0.57(0.28-1.14) | 0.6(0.31-1.15) |
| jugular vein thrombosis | 0.81(0.3-2.17) | 0.5(0.21-1.21) |
| mesenteric artery thrombosis | 1.16(0.29-4.66) | 0.74(0.1-5.29) |
| mesenteric vein thrombosis | 2.22(1.15-4.3) | 1.16(0.48-2.8) |
| pelvic venous thrombosis | 2.92(1.5-5.67) | 0.51(0.21-1.23) |
| peripheral artery thrombosis | 0.97(0.48-1.94) | 0.88(0.42-1.84) |
| portal vein thrombosis | 1.59(1.04-2.42) | 0.8(0.42-1.54) |
| postoperative thrombosis | 1.55(0.5-4.85) | 1.72(0.71-4.17) |
| pulmonary thrombosis | 1.04(0.75-1.43) | 0.83(0.62-1.11) |
| renal vein thrombosis | 0.5(0.07-3.57) | 0.36(0.05-2.59) |
| retinal vascular thrombosis | 3.17(1.3-7.72) | 2.86(1.42-5.79) |
| retinal vein thrombosis | 2.5(1.11-5.63) | 2.27(1.13-4.58) |
| superficial vein thrombosis | 0.8(0.4-1.6) | 0.3(0.14-0.63) |
| superior sagittal sinus thrombosis | 2.57(1.06-6.25) | 0.4(0.1-1.61) |
| thrombophlebitis | 2.5(1.72-3.64) | 1.31(0.87-1.95) |
| thrombophlebitis septic | 1.6(0.22-11.58) | 3.84(1.21-12.17) |
| thrombosis | 0.74(0.65-0.85) | 0.74(0.66-0.83) |
| thrombosis in device | 0.24(0.09-0.64) | 0.61(0.29-1.28) |
| thrombotic microangiopathy | 0.21(0.1-0.43) | 0.09(0.03-0.28) |
| thrombotic stroke | 0.69(0.17-2.78) | 0.28(0.04-2.01) |
| thrombotic thrombocytopenic purpura | 0.23(0.07-0.71) | 0.53(0.26-1.06) |
| vascular stent thrombosis | 0.09(0.01-0.64) | 0.26(0.04-1.82) |
| vena cava thrombosis | 1.23(0.58-2.6) | 0.61(0.25-1.47) |
| venous thrombosis | 0.95(0.55-1.63) | 1.17(0.76-1.8) |
| venous thrombosis limb | 0.49(0.19-1.32) | 0.73(0.36-1.46) |

*ROR*, reporting odds ratio; *CI*, confidence interval; *95% CI*, two-sided for ROR.

**Table S4** Statistical distribution data of induction time for thrombosis-related adverse events associated with five TNF-α inhibitors.

|  | **Time to induction of thrombosis-related adverse reactions** | | | |
| --- | --- | --- | --- | --- |
| **Drug** | **Average（days）** | **Median（days）** | **Upper quartile（days）** | **Lower quartile（days）** |
| **Adalimumab** | 555.92 | 222 | 61 | 724 |
| **Golimumab** | 703.05 | 332 | 86 | 1314 |
| **Certolizumab Pegol** | 481.23 | 273.5 | 70 | 709.25 |
| **Etanercept** | 1100.02 | 603.5 | 110.75 | 1516.25 |
| **Infliximab** | 534.32 | 165 | 35 | 729.5 |
| **All drugs** | 678.79 | 278 | 69 | 903 |

**Table S5** Statistical test for the induction time corresponding to five TNF-α inhibitors.

|  | **statistic** | ***p*-value** |
| --- | --- | --- |
| Adalimumab | 0.241^a^ | ＜0.001^b^ |
| Golimumab | 0.226^a^ | ＜0.001^b^ |
| Certolizumab Pegol | 0.195^a^ | ＜0.001^b^ |
| Etanercept | 0.272^a^ | ＜0.001^b^ |
| Infliximab | 0.272^a^ | ＜0.001^b^ |
| 5 TNF-α inhibitors | 100.945^c^ | ＜0.001^d^ |
| Infliximab-Adalimumab | 2.182^e^ | 0.291^f^ |
| Infliximab-Certolizumab Pegol | 1.684^e^ | 0.922^f^ |
| Infliximab-Golimumab | 2.689^e^ | 0.072^f^ |
| Infliximab-Etanercept | 8.792^e^ | ＜0.001^f^ |
| Adalimumab-Certolizumab Pegol | -0.401^e^ | ＞0.999^f^ |
| Adalimumab-Golimumab | -1.815^e^ | 0.695^f^ |
| Adalimumab-Etanercept | -8.682^e^ | ＜0.001^f^ |
| Certolizumab Pegol-Golimumab | 1.312^e^ | ＞0.999^f^ |
| Certolizumab Pegol-Etanercept | -4.523^e^ | ＜0.001^f^ |
| Golimumab-Etanercept | -1.747^e^ | 0.807^f^ |

^a^ Kolmogorov-Smirnov statistic *D*-value

^b^ Lilliefors-corrected Kolmogorov-Smirnov *p*-value

^c^ Kruskal­Wallis H Test statistic *H*-value

^d^ Kruskal­Wallis H Test *p*-value

^e^ Bonferroni-corrected Kruskal­Wallis H Test statistic *Z*-value

^f^ Bonferroni-corrected Kruskal­Wallis H Test *p*-value
